# Screening of the best time window for MSC transplantation to treat acute myocardial infarction with SDF-1α antibody-loaded targeted ultrasonic microbubbles: An *in vivo* study in miniswine

**DOI:** 10.1515/biol-2022-0620

**Published:** 2023-06-21

**Authors:** Lingjie Yang, Rong Hu, Chen Yuan, Lina Guan, Yuming Mu

**Affiliations:** Department of Echocardiography, First Affiliated Hospital of Xinjiang Medical University, Xinjiang Key Laboratory of Ultrasound Medicine, No. 137, Li Yu Shan South Road, Urmuqi 830011, China

**Keywords:** myocardial contrast echocardiography, targeted ultrasonic microbubbles, stem cell transplantation, myocardial infarction

## Abstract

The present study aimed to screen the best time window for the transplantation of bone marrow mesenchymal stem cells (MSCs) after acute myocardial infarction (MI) through targeted ultrasound microbubbles loaded with SDF-1α antibody. Thirty-six MI miniswine were randomly divided into six experimental groups according to the duration after infarction (1 day, 3 days, 1 week, 2 weeks, 3 weeks, and 4 weeks after infarction). MSCs were labeled with BrdU and then injected through the coronary artery in the stem cell transplantation group to detect the number of transplanted MSCs at different time points after MI. Three miniswine were randomly selected as the control group (sham operation: open chest without ligation of the coronary artery). All SDF-1α groups and control groups were injected with a targeted microbubble ultrasound contrast agent. The values of the myocardial perfusion parameters (*A*, *β*, and *A* × *β*) were determined. *A*_T_, *β*_T_, and (*A* × *β*)_T_ varied with time and peaked 1 week after MI (*P* < 0.05). The number of transplanted stem cells in the myocardium through coronary injection of MSCs at 1 week was the greatest and consistent with the changing tendency of *A*_T_, *β*_T_, and (*A* × *β*)_T_ (*r* = 0.658, 0.778, 0.777, *P* < 0.05). *β*_T_(*X*), (*A* × *β*)_T_(*X*), and the number of transplanted stem cells was used to establish the regression equation as follows: *Y* = 36.11 + 17.601*X*; *Y* = 50.023 + 3.348*X* (*R* ^*2*^ = 0.605, 0.604, *P* < 0.05). The best time window for transplanting stem cells was 1 week after MI. The myocardial perfusion parameters of the SDF-1α targeted contrast agent can be used to predict the number of transplanted stem cells in the myocardial tissue.

## 1 Introduction

Stem cells have widely been applied in basic research and clinical trials on the regenerative repair of myocardial damage. Recent studies have confirmed that the injection of bone marrow cells is safe and effective for patients with myocardial infarction (MI) or ischemia and that there are no local or general complications [1]. In addition, there is increasing evidence indicating that mesenchymal stem cells (MSCs) also highly transferred their mitochondria to effectively protect against ischemic heart disease [2], eye diseases [3], and lung injury [4]. However, according to Henning [5], the use of different kinds of transplanted cells, different methods of transplantation, different transplanting time, different length of follow-up visits, and different evaluation methods and evaluation parameters might contribute to different effects of transplantation. Some researchers believe that transplantation of stem cells 2 weeks or more after MI has a greater curative effect in limiting the infarction area and improving the left heart function [6]. However, other researchers [7] have shown that the curative effect of transplantation is best in the early stages of MI, namely, within 7 days after MI. Other studies [8] have shown that compared with the placebo group, transplanting stem cells at different time points causes no significant improvement in cardiac function. Therefore, to further optimize and improve the therapeutic effect of stem cells on MI, it is vital to explore the best transplanting time.

Stem cell homing plays an important role in the treatment of infarcted myocardium. Stromal cell-derived factor-1alpha (SDF-1α) is a chemokine that plays a critical role in stem cell homing. SDF-1α specifically anchors CXCR4 onto the surface of the stem cell membrane and activates the SDF-1α/CXCR4 pathway, which triggers bone marrow MSCs to home to the inflammatory tissue area [9]. Experimental and clinical studies have shown that SDF-1α is upregulated in the microenvironment of the MI area and regulates stem cell migration to sites of injury [10]. Thus, the concentration of SDF-1α in infarcted myocardium is an important factor affecting the homing of stem cells. However, it is very difficult to determine the expression of SDF-1α in the infarcted area noninvasively and *in vivo*. Therefore, to make better use of the role of transplanted stem cells in the treatment of MI, it is necessary to study the expression of SDF-1α in the infarcted myocardium and to determine the effect of changes on stem cell homing.

Ultrasound microbubbles are widely used as carriers in drugs or gene-targeted therapies. In addition, ultrasound microbubbles are also used to carry molecular ligands with specific expression into pathological tissues to achieve the purpose of contrast-enhanced ultrasound (CEU) molecular imaging by identifying targets of molecules abnormally expressed in a pathological situation [11]. CEU molecular imaging techniques that can provide unique information on ischemia, angiogenesis, vascular inflammation, and thrombus formation have been developed. In our previous research [12], we determined the dynamic expression of SDF-1α in an acute MI (AMI) swine model by ultrasound molecular imaging via targeted microbubbles. In this study, we further explored the value of ultrasonic molecular imaging in the evaluation of stem cell homing. SDF-1α-targeted microbubbles and myocardial contrast acoustic imaging were used in the miniswine MI models to explore the best time window for stem cell transplantation after MI by analyzing the correlation between the myocardial perfusion of the targeted contrast agent and the number of transplanted stem cells.

## 2 Materials and methods

### 2.1 Preparation of targeted ultrasound microbubbles

Targestar SA (Targeson, Inc., San Diego, CA, USA) is a contrast agent with a streptavidin-coated surface to allow one-step coupling of biotinylated ligands that are suitable for molecular ultrasound imaging. There are approximately 1 × 10^9^ microbubbles in each milliliter of the suspension. Targeted microbubbles that specifically bind with SDF-1α were prepared as previously described [13]. Briefly, streptavidin-coated microbubbles were incubated with biotinylated rabbit anti-swine SDF-1α monoclonal antibody (BIOSS, Inc., Beijing, China). Then, the suspension was incubated at room temperature and centrifuged at 400 rpm to wash out unbound unconjugated antibodies. Fluorescence microscopy and flow cytometry were used to evaluate the physical and chemical properties and the combined ratio of the targeted ultrasound microbubbles. The microbubbles that were not incubated with the antibody were used as the nontargeted microbubbles.

### 2.2 MI model

Eight-month-old adult miniswine (weight, 21.4 ± 1.55 kg) were provided by the Experimental Animal Research Center of Xinjiang Medical University. The miniswine were treated following the policies and regulations approved by the Animal Ethics Committee of the First Affiliated Hospital of Xinjiang Medical University. The animal research complied with all relevant national regulations and institutional policies for the care and use of animals.

Surgical procedures on the MI model were performed as previously described [12]. Briefly, the miniswine were anesthetized using ketamine (10–15 mg/kg, intramuscularly) and atropine (25 μg/kg, intramuscularly), and venous channels were established through the porcine ear vein using a bolster. During the operation, the swine were given intravenous anesthetics (ketamine hydrochloride and midazolam, 4.0 mg/kg), and suxamethonium chloride injection (0.2 mg/kg) before establishing mechanical breathing passages using tracheal intubation. The electrocardiogram, heart rate, and oxygen saturation were monitored using an electrocardiogram (ECG, USA, HP Cm XL+) monitor during the operation. An electrical defibrillation instrument was used for emergencies. Three tablets of Betaloc were applied after anesthesia was induced, and an intravenous drip of lidocaine (at a speed of 50 μg/kg per minute) was used to prevent ventricular fibrillation and maintain the heart rate at 60–100 bpm. Thoracotomy and ligature of the left anterior descending coronary artery were initiated according to aseptic processing. The thorax was opened along the fourth intercostal space at the left edge of the sternum, and the pericardium was cut to expose the heart. From the starting point of the left anterior descending artery to the apex of the heart, ligation was conducted in the middle, that is, at 50% of the distance. The slipknot was released after 50 min. The chest was closed with suture layer by layer, and ECG monitoring was continued for 30 min after the operation. All animals received analgesics (buprenorphine: 0.3 mg twice a day) and antibacterial treatment (penicillin: 2400 IU) within 3 days after the operation. Preoperative and postoperative ECG and echocardiogram were used in all of the experimental animals, and serum myocardial enzymes were monitored within 24 h after the operation.

**Figure 1:**
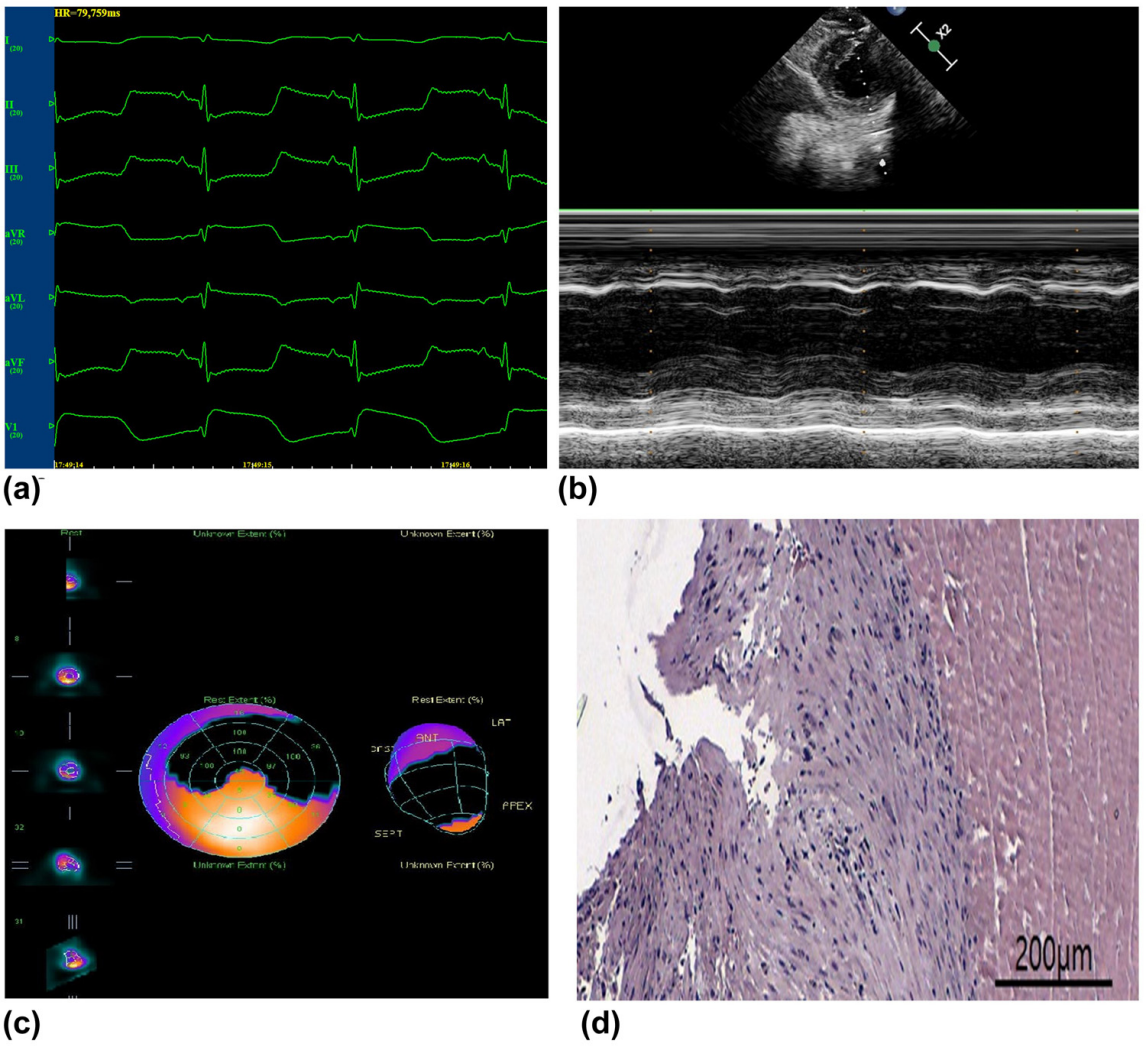
Confirmation of the acute MI model. (a) ECG: ST segments increased by >0.5 mV in the chest leads. ECG, electrocardiogram. (b) Ultrasonic cardiograms: Segmental weakening of left ventricular wall motion on motion mode echocardiography. (c) ECT: Local defects and/or decreases were observed in the radioactive signal. (d) Pathology: The infarcted myocardium showing edema, neutrophil infiltration, coagulative necrosis, and nuclear degeneration. Bar = 200 μm.


The MI model was confirmed by the following tests: ECG, emission computed tomography, pathology, and echocardiography (Figure 1).

**Ethical approval:** The research related to animal use has complied with all the relevant national regulations and institutional policies for the care and use of animals, and has been approved by the Animal Ethics Committee of the First Affiliated Hospital of Xinjiang Medical University.

### 2.3 Animal grouping

Three of the 39 miniswine were randomly selected as the control group (*n* = 3, sham operation group: open chest without ligation of the coronary artery). The remaining 36 MI model miniswine were randomly divided into six groups as the experimental group according to the duration after infarction (1 day, 3 days, 1 week, 2 weeks, 3 weeks, and 4 weeks after infarction). In the experimental group, MSCs were injected through the coronary artery to detect the number of transplanted MSCs at different time points after MI. All of the groups were injected with the nontargeted ultrasound contrast agent (T + C group) or targeted microbubble ultrasound contrast agent (T + T group) successively.

### 2.4 Real-time myocardial contrast echocardiography

Myocardial contrast echocardiography was performed. Briefly, a Philips IE-33 (Philips Electronics, Andover, MA, USA) ultrasound imaging instrument with an S5-1 cardiac probe was used with a frequency in the range of 2–6 MHz. After miniswine were anesthetized, 1 mL of the nontargeted contrast agent was injected through the marginal ear vessel. In the parasternal short-axis view of the left ventricular papillary muscle, scintigraphy was triggered when myocardial imaging was stable, and a high mechanical index pulse was initiated to completely and instantly destroy the microbubbles in the myocardium. Then, the instrument automatically switched to the low mechanical index and recorded the dynamic image of the entire process until the contrast agent was cleared. Thirty minutes after the contrast agent was completely cleared, the ultrasound parameters were kept unchanged, 1 mL of the targeted ultrasound microbubbles contrast agent was injected, and the above steps were repeated.

**Figure 2:**
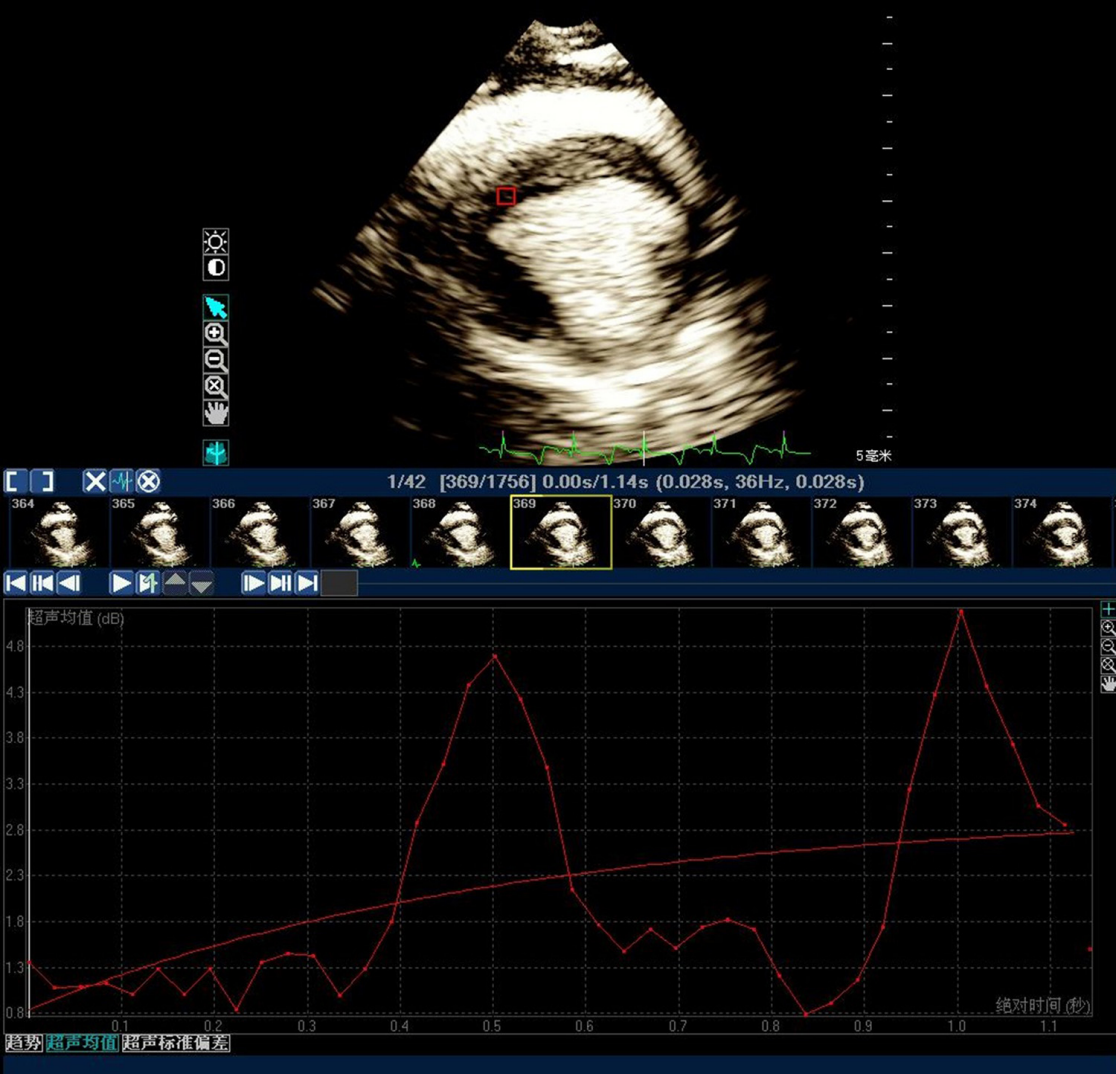
Myocardial contrast echocardiography. After injecting the microbubble contrast agent *in vivo*, the acoustic signal intensity of the MI area was detected.

Q-Lab software was used to obtain the myocardial perfusion parameters*. A* is the myocardial blood volume, *β* is the myocardial blood flow velocity, and *A* × *β* is the myocardial blood flow volume (Figure 2).

### 2.5 Stem cell transplantation and detection

MSCs, which were provided by the Xinjiang Medical University Laboratory (Xinjiang, China), were extracted from the bone marrow of miniswine and identified by flow cytometry as CD44/CD29-positive and CD45-negative. The cells were cultured with Dulbecco’s modified Eagle’s medium (DMEM) (Hyclone, Logan, UT, USA) containing 10% fetal bovine serum (FBS) (Gibco, Thermo Fisher Scientific, Waltham, MA, USA) at 37°C in 5% CO_2_. MSCs were transplanted at different time points after infarction (1 day, 3 days, 1 week, 2 weeks, 3 weeks, 4 weeks) according to the grouping.

Before each transplantation, MSCs were labeled with 5-bromo-2-deoxyuridine (BrdU; Invitrogen). In brief, the third-generation MSCs were incubated in the culture medium containing BrdU (10 µmol/L) to label MSCs 48 h before transplantation. Then, BMCs were harvested and washed three times with heparinized saline, and the cells were resuspended with heparinized saline to prepare cell suspension (1 × 10^8^ cells/mL). Cell transplantation was performed via the intracoronary administration route [14] using 4–6 fractional infusions parallel to balloon inflation over 2–4 min of 5 mL of cell suspension (1 × 10^8^ cells/mL). All cells were infused directly into the infarcted zone through the infarct-related artery via an angioplasty balloon catheter, which was inflated for approximately 2 min to block the coronary blood flow. This prevented the backflow of cells and produced stop flow beyond the site of balloon inflation to maximize the time of cells staying in the microcirculation area of the infarct-related artery and facilitate high-pressure infiltration of cells into the infarcted zone. Heparinization and filtration were carried out to prevent cell clotting and microembolization during intracoronary transplantation. Angiography was performed after intra-arterial cell transplantation to evaluate the blood flow of the artery.

Three hours after transplantation, the myocardial tissue was collected after euthanasia, fixed with 4% paraformaldehyde, and embedded. Immunohistochemical staining of BrdU was conducted following the manufacturer’s protocol (BrdU staining kit, Invitrogen). Briefly, the prepared paraffin-embedded sections of the myocardium were fixed in acetone and incubated with 10% goat serum for 30 min. The sections were then incubated with anti-BrdU monoclonal antibody (1:100, Invitrogen, Thermo Fisher Scientific) at 4°C overnight. The tissues were washed extensively in phosphate buffer saline (PBS), and detection was performed using a horseradish peroxidase-conjugated secondary antibody followed by colorimetric detection using a DAB (3,3′-diaminobenzidine) kit. The tissue was counterstained with hematoxylin (Sigma) and dehydrated with ethanol and xylene. The nuclei were stained brown in positive cells, and the positively stained area was counted under an Olympus IX70 light microscope (Olympus Inc., Melville, USA) to observe the transplanted MSCs in the myocardial tissue at the border of the infarction. Buffy BrdU represented positively labeled cells to show transplanted MSCs, and the number of transplanted stem cells was counted from the analysis of at least five randomly selected areas from each slice.

### 2.6 Statistical analysis

All data are presented as the mean ± SD. The data were analyzed using IBM SPSS statistics version 23.0 (SPSS, Inc., Chicago, IL, USA). The differences in the transplanted stem cell number among the groups were evaluated using one-way ANOVA. A *t*-test was used for comparisons between the two groups. Pearson correlation was used to analyze the correlations between the transplanted stem cell number and the myocardial perfusion parameters, and the level of *P* < 0.05 was considered statistically significant.

## 3 Results

### 3.1 Transplantation of MSCs

**Figure 3:**
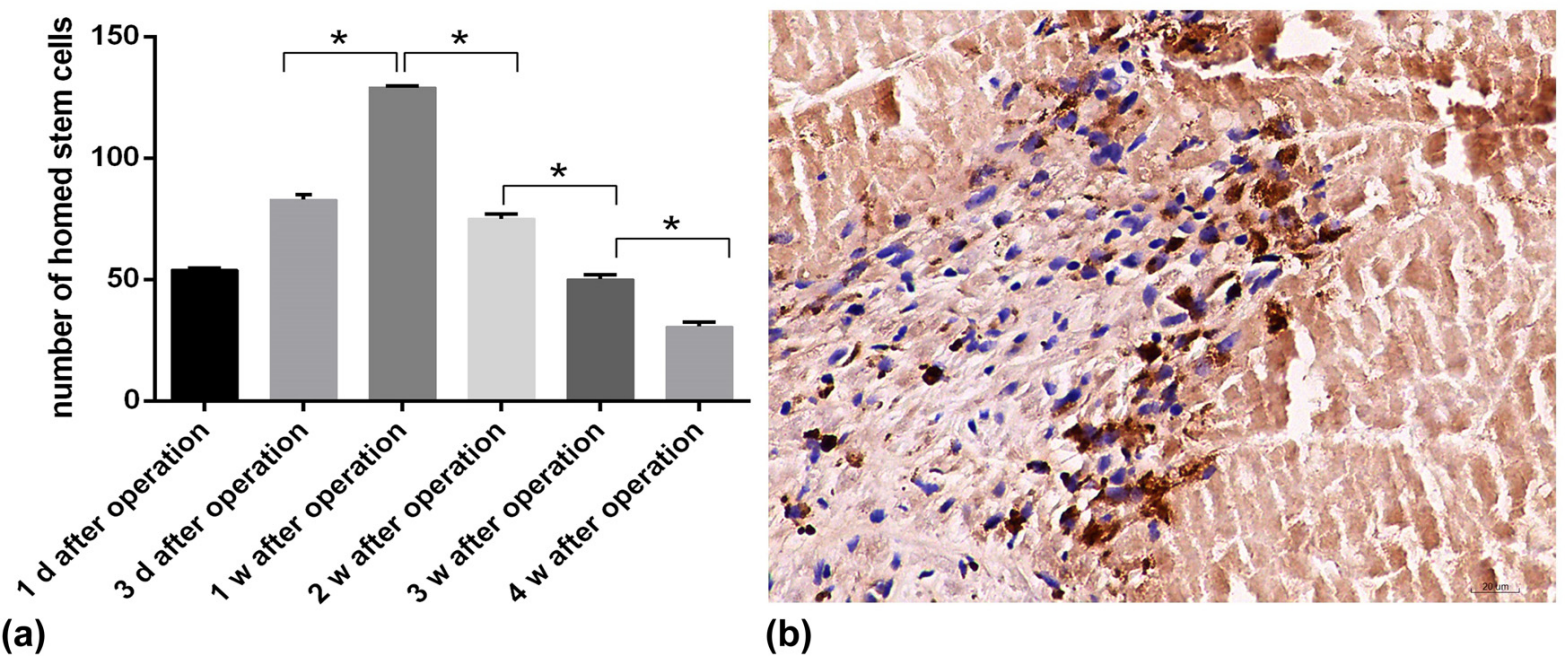
(a) Number of MSCs in myocardial tissue with cell transplantation at different time points after MI. (b) Representative image of BrdU immunohistochemical staining in the stem cell transplantation group at 1 week after MI. The positive cells labeled with BrdU are brown and show transplanted MSCs. Bar = 20 μm; *P < 0.05 vs other groups.

On day 1, day 3, week 1, week 2, week 3, and week 4 after the MI, the cell nuclei took on a Buffy BrdU marker of transplanted cells. The results showed that the number of transplanted cells was the greatest and the most concentrated at 1 week after MI (*P* < 0.05), as shown in Figure 3.

### 3.2 Correlation analysis of the myocardial perfusion parameters and the number of transplanted stem cells

**Figure 4:**
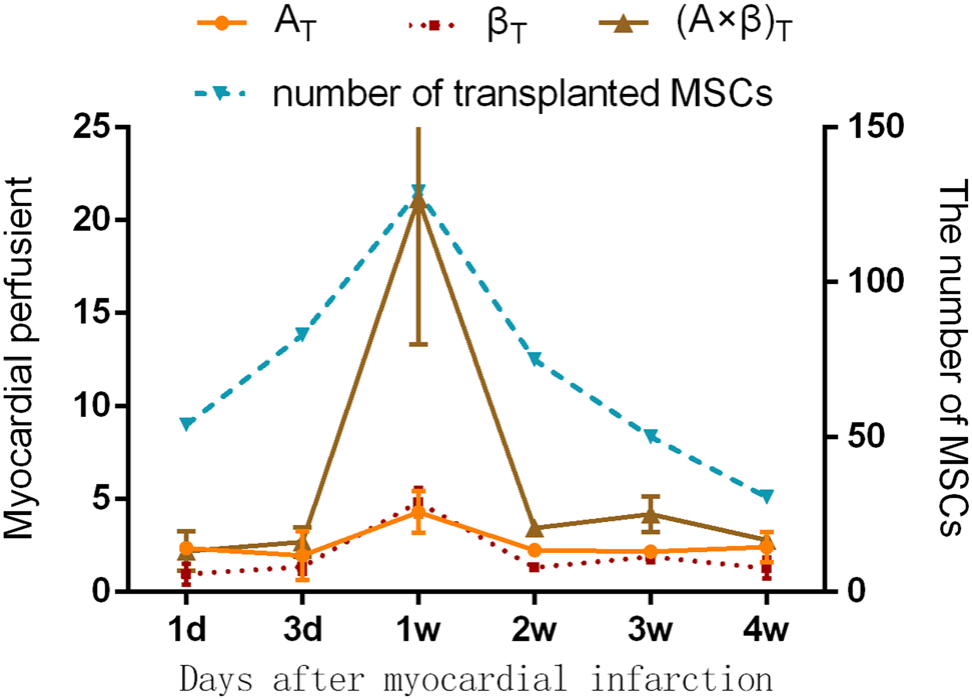
The changing tendency of myocardial perfusion parameters of the SDF-1α-targeted contrast agent and the number of transplanted stem cells in the myocardium at six different time points.

Changes in the myocardial angiography parameters were analyzed in our previous study [12]. The myocardial perfusion parameters *A*_T_, *β*_T_, and (*A* × *β*)_T_ of the targeted contrast agent varied with time but were consistent with the changing tendency of the number of transplanted stem cells in the myocardium at different time points after infarction. All of the parameters peaked 7 days after the infarction, as shown in Figure 4.

We showed the correlation between the myocardial perfusion parameters *A*_T_, *β*_T_, and (*A* × *β*)_T_ of the targeted contrast agent and the number of transplanted stem cells (*P* < 0.05). However, there was no correlation between the myocardial perfusion parameters *A*_c_, *β*_c_, and (*A* × *β*)_c_ of the nontargeted contrast agent and the number of transplanted stem cells, as shown in Table 1. *β*_T_(*X*), (*A* × *β*)_T_(*X*) and the number of transplanted stem cells were used to establish the regression equation as follows: *Y* = 36.11 + 17.601*X*; *Y* = 50.023 + 3.348*X* (*R* ^*2*^ = 0.605, 0.604, *P* < 0.05).

**Table 1:** Correlation analysis of myocardial perfusion parameters and the number of stem cells homing *r*(*P*)

Contrast agent	Myocardial perfusion parameters	*n*	*r*	*P*
Non-targeted	*A*_c_ (dB/s)	18	0.292	0.239
	*β*_c_ (s^−1^)	18	0.366	0.135
	(*A* × *β*)_c_ (dB/s)	18	0.499	0.035
Targeted	*A*_T_ (dB/s)	18	0.658	0.003
	*β*_T_ (s^−1^)	18	0.778	0.000
	(*A* × *β*)_T_ (dB/s)	18	0.777	0.000

## 4 Discussion

SDF-1α plays a significant role in the repairing of myocardial injury and promotes stem cell mobilization, migration, and survival. SDF-1α is expressed at different levels at different time points after MI. In this study, SDF-1α-targeted ultrasound microbubbles were prepared, and ultrasound molecular imaging was applied for the evaluation after cell transplantation. The research adopted reperfusion modeling after MI in miniswine, simulating clinical treatment of percutaneous coronary artery angioplasty as early as possible for patients with coronary artery disease who suffered MI, and stem cell transplantation was then performed. The results showed that after intracoronary injection of MSCs, the number of transplanted cells in the damaged myocardial area was the greatest on the seventh day after MI (*P* < 0.05), and the myocardial perfusion parameters of SDF-1α-targeted microbubbles correlated with the number of transplanted stem cells.

MSCs transplanted into damaged myocardial tissue are regulated by many factors, such as the SDF-1α/CXCR4 signal axis, intracellular signal pathway, adhesion molecules, and proteases. It has been reported that these factors are related to the mechanism of stem cell homing and heart repair. Among many inflammatory cytokines, SDF-1α has a stronger dose-dependent chemotaxis on stem cells and plays a protective role in limiting stem cell apoptosis, so it attracts much attention [15]. The content of SDF-1α is closely related to the homing of stem cells. Through the specific binding of antigen and antibody, analyzing the myocardial perfusion curve of microbubbles carrying anti-SDF-1α antibodies can indirectly indicate the expression of SDF-1α in the MI area. Based on the results of this study, the myocardial perfusion parameters of targeted microbubbles showed a unimodal curve change after MI and peaked at 7 days after MI. This trend was consistent with the changes in the number of transplanted stem cells in the infarcted myocardium at different time points after MI, and there was a correlation between the two. However, there was no correlation between the changes in the myocardial perfusion parameters of nontargeted microbubbles and the number of transplanted stem cells. The results confirm that SDF-1α-targeted ultrasound microbubbles can achieve noninvasive measurement of myocardial SDF-1α *in vivo* and guide the optimal timing of stem cell transplantation to ensure stem cell homing as much as possible, which shows great potential for screening MI patients before stem cell transplantation. In addition, bone marrow-derived MSCs were used in our study. Rigorous quality control is critical to reducing the batch-to-batch variation in MSC products. Recently, induced pluripotent stem cells-derived MSCs (iPSC-MSCs) [16] have displayed a higher proliferative capacity. Li et al. demonstrated that iPSC-MSCs were superior to bone marrow-derived MSCs in anti-apoptotic/pro-proliferative capacity through paracrine secretion of SCF and TGF-β1/2/3 [17,18]. Thus, iPSC-MSCs may become an alternative resource for bone marrow-derived MSCs in the future [19].

Targeted ultrasound contrast agents have the advantages of high specificity, good repeatability, and noninvasiveness. Compared with other existing quantitative research methods, this research method can monitor the expression of SDF-1α *in vivo*, noninvasively, and in real time. The disadvantage is that the damaged myocardial tissue and other tissues and organs cannot be assessed at the same time, and it is impossible to assess other inflammatory factors at the same time by one-time myocardial contrast echocardiography. In addition, targeted microbubbles are less stable under high shear forces, and these issues remain to be further explored in future targeted ultrasound contrast agent studies [20].

The limitations of this study are as follows: this study only analyzed the optimal time window for MSC transplantation to damaged myocardium from the perspective of SDF-1α/CXCR4 and did not consider other factors that might affect the number of homed stem cells. Furthermore, there may be some intra-arterial cell aggregation of transplanted cells by coronary injection, and the intratissue location of the transplanted cells needs to be determined. Immunosuppressants are required before and after cell transplantation *in vivo* [21] but, in our study, we did not use immunosuppressant agents after MSC transplantation, so the loss of BMSCs caused by immune rejection could not be excluded.

## 5 Conclusions

The concentration of SDF-1α in the infarcted myocardium is an important factor affecting the homing of stem cells. In this study, targeted microbubbles carrying SDF-1α antibody were used to explore the best time window for stem cell transplantation after MI. By observing the contrast ultrasound parameters of SDF-1α-targeted microbubbles and the number of transplanted MSCs after transplantation, the results show that the best time for transplantation of MSCs is 1 week after acute MI. With the establishment of the regression equation, our study confirmed that the SDF-1α-targeted contrast agent can be used to predict the number of transplanted stem cells after transplantation in the myocardial tissue, providing a theoretical basis for subsequent research on stem cell transplantation.
